# Analysis of free text in electronic health records for identification of cancer patient trajectories

**DOI:** 10.1038/srep46226

**Published:** 2017-04-07

**Authors:** Kasper Jensen, Cristina Soguero-Ruiz, Karl Oyvind Mikalsen, Rolv-Ole Lindsetmo, Irene Kouskoumvekaki, Mark Girolami, Stein Olav Skrovseth, Knut Magne Augestad

**Affiliations:** 1 Norwegian Centre for E-Health Research, University Hospital of North Norway, Norway; 2 Department of Statistics, University of Warwick, United Kingdom; 3 Department of Signal Theory and Communications, University Rey Juan Carlos, Spain; 4 Department of Mathematics and Statistics, UiT The Arctic University of Norway, Norway; 5 Department of Gastrointestinal Surgery, University Hospital of North Norway, Norway; 6 Department of Systems Biology, Technical University of Denmark, Denmark; 7 Department of Mathematics, Imperial College London, Exhibition Road, London, United Kingdom; 8 The Alan Turing Institute, British Library, 96 Euston Road, United Kingdom; 9 Department of Gastrointestinal Surgery, Akershus University Hospital, Oslo, Norway

## Abstract

With an aging patient population and increasing complexity in patient disease trajectories, physicians are often met with complex patient histories from which clinical decisions must be made. Due to the increasing rate of adverse events and hospitals facing financial penalties for readmission, there has never been a greater need to enforce evidence-led medical decision-making using available health care data. In the present work, we studied a cohort of 7,741 patients, of whom 4,080 were diagnosed with cancer, surgically treated at a University Hospital in the years 2004–2012. We have developed a methodology that allows disease trajectories of the cancer patients to be estimated from free text in electronic health records (EHRs). By using these disease trajectories, we predict 80% of patient events ahead in time. By control of confounders from 8326 quantified events, we identified 557 events that constitute high subsequent risks (risk > 20%), including six events for cancer and seven events for metastasis. We believe that the presented methodology and findings could be used to improve clinical decision support and personalize trajectories, thereby decreasing adverse events and optimizing cancer treatment.

For cancer patients who tend to have numerous encounters with the healthcare system, personalized care has become increasingly important^1^. However, identifying the information needed for high quality care and the interpretation of the frequently complex medical information that comes with patients, is a resource-consuming task^2^. Similarly, with increasing health care costs, inadequate resources, and a lack of a methodology for measuring outcomes and quantifying risks, the demand for interventions to improve health care delivery has gained considerable momentum from taxpayers, health care providers and policy makers^3^. For example, 30-day readmissions cost the National Health Service (NHS) in the United Kingdom alone 1,6 billion GBP per year^4^,^5^,^6^ and as hospitals observe an increased rate of adverse events, with unwanted consequences for the individual patient, there has never been a greater need to improve evidence-led medical decision-making^5^,^7^.

For cancer patients, clinical decisions must be tailored so that necessary treatment is provided in due time to avoid a more severe cancer development. In the case of cancer, a variety of factors may influence diagnosis, treatment, and ultimately survival. Understanding the conditions and variations that affect outcomes is therefore, important for decision-making and delivery of high quality care^8^. Ultimately, the treatment profile of the individual cancer patient must be tailored to the patient’s unique treatment profile and expected trajectory^9^.

The concept of trajectories has existed for some time in the healthcare domain. For example, Jensen *et al*.^10^ converted disease observations for patients over a 15 years period to disease trajectories. Ebadollahi *et al*.^11^, predict patient trajectories from physiological data using temporal trends to monitor if a patient will experience a subsequent event, while Ji *et al*.^12^ used social health records to develop prediction models for comorbidity relationships and condition trajectories.

However, to the best of our knowledge, no previous studies describe the creation of trajectories directly from free text. It is important to emphasize that still nowadays a lot of the patient information in the EHR is in free text. Free text is ubiquitous as it is used to keep track and record of the health of patients and serves as communication between healthcare providers. For this reason, unstructured EHR text may provide complete descriptions that would not have been possible to obtain from data in a structured form^13^.

The present paper focuses on identifying cancer patient trajectories in need of resource demanding treatment and repeated hospital admissions, and - empowered by control of confounders - identifying events that enable each patient’s risk of subsequent events (i.e. signs, symptoms, treatment choices, surgery, death) to be estimated from the free EHR text.

Towards this, we used the Norwegian version of Medical Subject Headings (NOR-MeSH)^14^ which has Norwegian terms and synonyms for diseases, drugs and surgical procedures that allow for automated data capture. In comparison, ICD-10^15^ is a disease classification system and the ATC codes^16^ is an anatomical classification system for drugs.

The threats to causal inference are different in experimental and observational studies and in observational settings the comparability between exposure and negative control may only be approximated. Therefore, we grouped non-causal associations into three categories; inaccurate measurements (recall bias), selection bias, and confounders^17^. With this we provide a full assessment of predictive variables that by control of confounders may be used to improve cancer care and provide tailored treatment.

In statistics, a confounder is an extraneous variable in a statistical model that correlates with both the dependent and the independent variable, in a way that explains partly or fully the correlation between these two variables. For example, the argument presented by R.A. Fisher against R. Doll and A.B. Hill’s initial work on the link between smoking and lung cancer was that there might be a confounder such as a genetic predisposition to lung cancer and smoking^18^.

We hypothesized that it is possible to identify the most common patient trajectories of our cohort of cancer patients from free text and identify the most common trends among our patients. Secondly, we evaluated the trajectories as predictors for subsequent events (i.e. signs, symptoms, treatment choices, surgery, and death) that are associated with cancer.

Towards this, we analyzed two cancer trajectory sets, the “disease trajectories” and the “event trajectories”. The disease trajectory was created to describe how patients progress from symptoms to diseases and, eventually, death, and was used to identify the most common cancer trajectories. The “event trajectory” consisted of symptoms, disease events, admission type, medication events, surgical procedures, and was used to predict the sequential events associated with our patients.

## Results

### Ethics and data description

The Data Inspectorate and the Ethics Committee at the University Hospital of North Norway (UNN) approved the study together with the data management protocol. Our cohort was identified as all patients undergoing a gastrointestinal surgical procedure (belonging to Chapter J in Nomesco Classification of Surgical Procedures) at UNN in the years 2004–2012. For these patients, we have records from 1999, when the EHR system was introduced to 2014, where we retrieved the records.

From the list of 7,741 patients, we extracted the corresponding free text documents from the EHRs. The main patient journals, i.e., the admission journal, nurse notes, doctor notes, descriptive surgical reports, intensive care reports and the discharge note were retrieved together with the date of death from the Norwegian Death Registry.

In total, we have 1,133,223 unstructured EHR text documents, including 77,445 admission reports and 2,781 intensive care unit reports that describe our cohort of 7,741 patients.

### Conversion of EHR texts into conceptual information

To conceptualize the Norwegian EHR text, we developed a decompounder that splits Norwegian compounded words into longest possible matches to the terms and synonyms in NOR-MeSH.

In this setup, words that are too long will not provide sufficient mapping, and splits that are too short will introduce noise. For this reason, splits have both lower and upper boundaries. Supplementary Figure S1 shows the evaluation of the minimum length of a split of compounded words in word characters. By manually looking at the number of correctly and incorrectly decomposed words we found that a minimum split no shorter than five characters provided the most accurate decomposition (accuracy, 80%).

Since written language comes with a certain degree of flexibility, the unstructured EHR text was conceptualized (marked with the position of each match) by matching terms and synonyms from NOR-MeSH to the text corpus of the EHR system, using the Smith-Waterman matching algorithm^19^ to allow words and concepts to be matched flexibly.

Supplementary Figure S1 illustrates the alignments between corpus words and those of our NOR-MeSH terms. Here, the scores of the alignments depend on the lengths of the strings compared. We observed that correct alignments cluster above the score of those that are invalid. Therefore, we drew a plane (illustrated as a line) as cutoff for accepted mappings as presented in Supplementary Figure S1.

We further evaluated the accuracy of the number of gaps considering correctly and incorrectly matched terms. Supplementary Figure S1 illustrates that allowing no more than two gaps in an alignment produced the most accurate mapping (accuracy, 85%).

The matches were curated and evaluated manually by computing the number of correctly and incorrectly matched terms. We estimated that we mapped the terms with an accuracy of 92% and a fall-out (false positives) of 5%.

We distinguished diseases, drugs and symptoms with the NOR-MeSH hierarchy, with 2,245 concepts for disease and symptoms, 1,936 concepts for drug and medications, and 301 concepts for surgical procedures.

### Acquiring patient data in context

As clinical decisions are often derived from information about a patient’s status and a patient’s medical history, the terms we are interested to retrieve are terms that describe patient events that occur now (real-time/current), and terms that describe a patient’s past (retrospective/history). Thus, we considered patient information in EHRs to consist of three main types of descriptions: (1) real-time data, (2) retrospective data, and (3) negated findings and correspondences, such as internal communication (noise). When mapping the terms and synonyms of diseases, drugs and surgical procedures in NOR-MeSH to the text corpus of EHR’s, a considerable amount of terms and synonyms will be mapped in text not directly describing the state of the patient, such as negated findings and correspondences and internal communication (noise).

We then trained three Naïve Bayes classifiers: one for disease and symptoms, one for drugs and medication and one for the surgical procedures^20^,^21^,^22^ and we compiled language features for each of the three classifiers, to distinguish between real-time events and noise (current – noise), retrospective events and noise (history – noise) and real-time events and retrospective events (current – history).

The classifier for diseases and symptoms recognition was trained with forward selection of features and with 10-fold cross validation. The classifier could accurately recognize concepts of diseases and symptoms with an F1-score^23^ of 0.83 using 103 language features (current – noise, F1 = 0.83, history – noise, F1 = 0.85, current–history, F1 = 0.82) where in this setup negations and negated findings were recognized by the classifier.

The classifier for drug and medication identification was trained and evaluated likewise and could correctly recognize concepts of drugs and medications with an F1 score of 0.80 using 138 language features (current – noise, F1 = 0.83, history – noise, F1 = 0.74, current – history, F1 = 0.84).

Lastly, the classifier for surgical procedures could correctly recognize concepts with an F1 score of 0.83 using 114 language features (current – noise, F1 = 0.79, history – noise, F1 = 0.82, current – history, F1 = 0.87).

**Figure 1:**
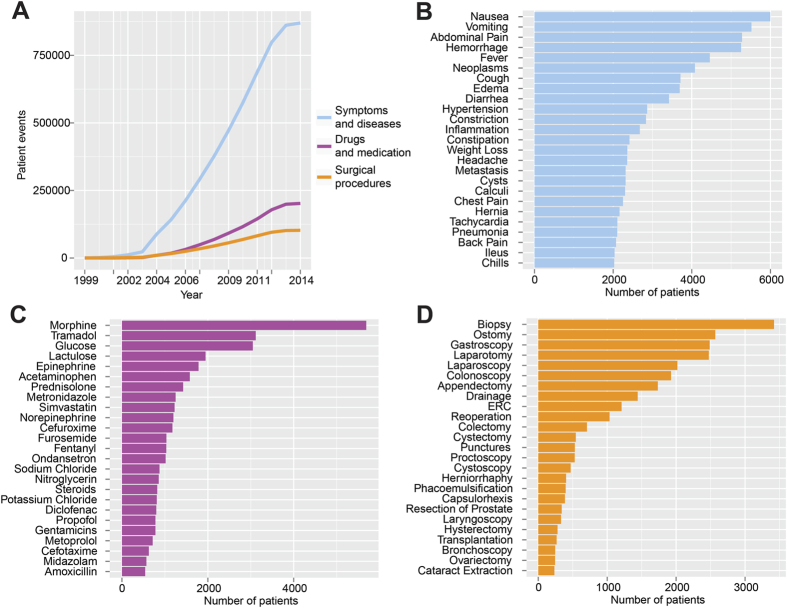
Information stored in free text of EHRs. (**A**) Patient events in terms of symptoms and diseases (blue), drugs, medication (magenta) and surgical procedures (orange) accumulated over time in the EHRs. (**B**) The most common symptoms and diseases. (**C**) The most common drugs and medication and (**D**) the most common surgical procedures.

The amount of information learned from the EHRs naturally increased with time (Fig. 1A). The number of learned observations and events that were related to diseases and symptoms was far greater than those for drugs (4.3 times) and surgical procedures (8.5 times) with 869,731 disease and symptom observations, 201,990 drug and medication events and 102,561 surgical procedure events. Therefore, the clear majority of the free text in the EHRs represents a rich archive of symptom and disease observations supported by drug, medication and surgical procedure descriptions. Figure 1B illustrates the 25 most common diseases and symptoms, with nausea (5,996 patients), vomiting (5,516 patients), abdominal pain (5,277 patients), hemorrhage (5,253 patients) and fever (4,458 patients), as the most common symptoms, and cancer (4,080 patients) as the most common disease observation. Similarly, the most common drugs and medications used (Fig. 1C) included morphine (5,689 patients), tramadol (3,116 patients), intravenous glucose (3,048 patients) lactulose (1,946 patients) and epinephrine (1,783 patients). Regarding surgical procedures (Fig. 1D), it was unsurprising that the simple procedure of biopsy (3,420 patients) ranked as the most common, followed by stoma formation (2,568 patients), upper endoscopic procedures (2,486 patients) and laparotomy (2,473 patients).

Using the non-parametric Kolmogorov-Smirnov test^24^, we evaluated whether the occurrences of symptoms and diseases among our patients depends on age. We found that although few diseases and symptoms appeared earlier or later in life than others (Supplementary Figure S2), the variation proceeded non-significant differences (p-value > 1.5e-4), when correction for multiple testing with the Bonferroni method and a 25% level of significance enables even small variations in age to pin out^25^.

### Construction of patient trajectories

Our goal was to identify common cancer patient trajectories, within the trajectory framework described by Murray *et al*.^9^. The typical cancer trajectory entails a predicable decline in physical health over a period of weeks to several years. Our patient population consists of patients with colorectal cancer, pancreatic cancer, ventricular cancer and esophageal cancer. In general, these patients usually have a short life expectancy; for instance, approximately 40% of the patients with colorectal cancer will experience recurrent cancer disease within five years^26^. In addition, patients are frail, with numerous concomitant chronically diseases, like obstructive pulmonary disease, hearth failure, dementia etc., i.e. diseases that are negatively affected by the progressing cancer disease.

For the trajectories to be clinically meaningful, they had to be composed of patient events that appeared together (i.e. notes related to hospital admissions, readmissions, adverse events, surgeries, medications, treatment decisions among others). Therefore, the construction of trajectories from Frequent Item Set (FIS) mining^27^ is obvious because medical events that repeatedly appear together are likely to have a clinically meaningful relationship.

**Figure 2:**
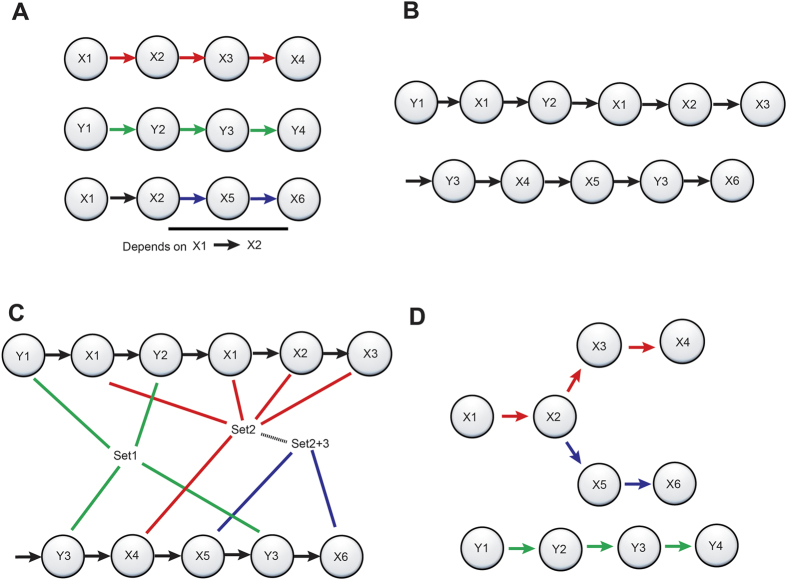
Construction of trajectories. (**A**) Progression of health state to multiple morbidities X and Y. (**B**) Variations in how patient information is registered yields a distorted information space observed by clinicians. (**C**) Frequent Item Set (FIS) mining identified observations that repeatedly appear together. (**D**) Trajectories are created using order of first appearance.

The construction of the trajectories has been summarized in Methods and further illustrated in Fig. 2A–C. Thus, we identify two sets of sub trajectories within the cancer trajectory framework, while reserving data from a set of 1,000 random patients for later validation (referred to as our external validation set), i.e.:

1. The disease trajectories. Symptoms and disease observations together with information from the Norwegian Death Registry (i.e. symptom - disease - death). This trajectory set was created to describe how patients progress from symptoms to diseases and, eventually, death.
2. The event trajectories. Symptoms, disease events, admission type, medication events, surgical procedures and information from the death registry (symptom → disease → admission → drug → surgery → death). This trajectory set was created to exhaustively quantify risk from the EHR text.

From 2,344,684 trajectories, we found 213,675 trajectories for subsequent events with a risk of at least 10% (20%, 123,591 trajectories; 30%, 53,510 trajectories; 40%, 13,776 trajectories; 50%, 2,033 trajectories; 60%, 157 trajectories; and 70%, two trajectories). The full list of raw data trajectories is included in Supplementary Dataset S1. Most of the trajectories led to an increased risk of a subsequent symptom or disease (74%), while some trajectories led to increased risks of medication (23%) or surgery (3%).

### Evaluation of trajectories in reconstructing patient events

The method for constructing trajectories depends on the questions the latter are expected to answer. For a patient experiencing events a, b, c and d, we used trajectories with events a and b to predict c, and trajectories with a, b and c to predict d. We evaluated the trajectories’ ability to reproduce the patient data by applying them step-wise forward on our external data set.

**Figure 3:**
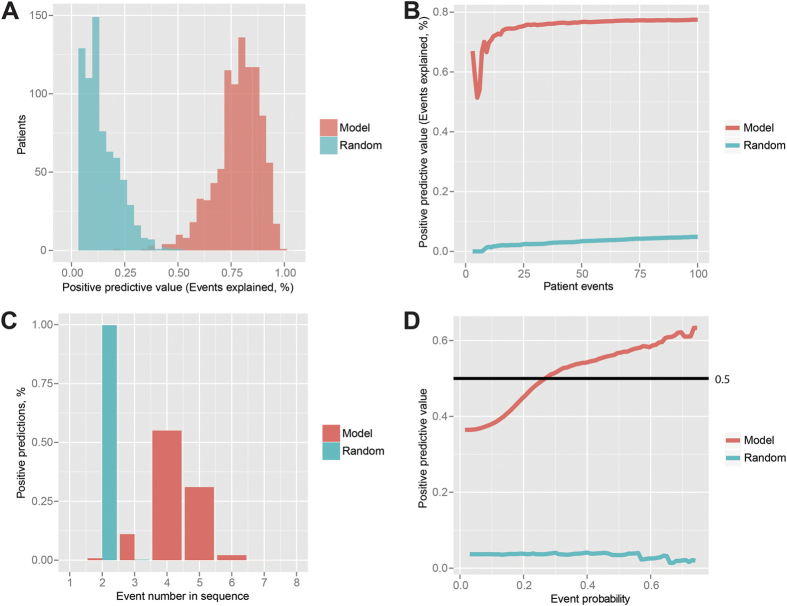
Reconstruction of patient events ahead in time. (**A**) The positive predictive value (PPV) (events explained, %) by trajectories compared with randomized trajectories of the same size. (**B**) The PPV in terms of the number of events known for a patient. (**C**) Positive predictions, % in terms of the event sequence number in the trajectories. (**D**) PPV in terms of event probability.

For our patients, we have data from the first encounter and an average of 63 months (~5.3 years) ahead with an average of 142.6 events. Figure 3A demonstrates that our trajectories could predict upcoming events in our external patient data set with a positive predictive value (PPV) of 80%. In comparison, randomized trajectories, where sequence of events and length has been randomized (control group), have a PPV of 19.4%.

Figure 3B illustrates that to estimate 75% of the upcoming events we needed approximately 20 unique known events. The EHRs do contain some repeated events; nevertheless, for 25% of the patients, 20 unique events were recorded within two weeks (15 days) from the first encounter, and for 50% of the patients within one year (394 days) from the first encounter.

As the trajectories have different numbers of events in sequence, we sought to determine how many events in the sequence of events produce the most predictions that match our external data set (our positive predictive paths). Figure 3C reveals that the trajectories produced the most positive predictions at event number 4 (55% of the predictions) and 5 (31% of the predictions) in the sequence of events. In comparison, randomized trajectories (~99%) produced nearly all predictions at event number 2. Therefore, randomized trajectories are not likely to predict any events beyond second order of appearance.

Finally, we evaluated the PPV for the probabilities on the trajectories moving from one event to the next along the trajectory and compared them with those of the randomized trajectories (Fig. 3D). As expected, we found that the PPV of the trajectories (model) increased with event probabilities, while the PPV of the randomized model remained constantly low.

### Measuring health changes

For patients with longer medical histories and repeated admissions, such as cancer patients, clinical decisions often must be tailored so that necessary care is provided in due time to avoid further complications. By understanding the health changes, we learn the characteristics of who may have adverse events and what their downstream complications may be.

**Figure 4:**
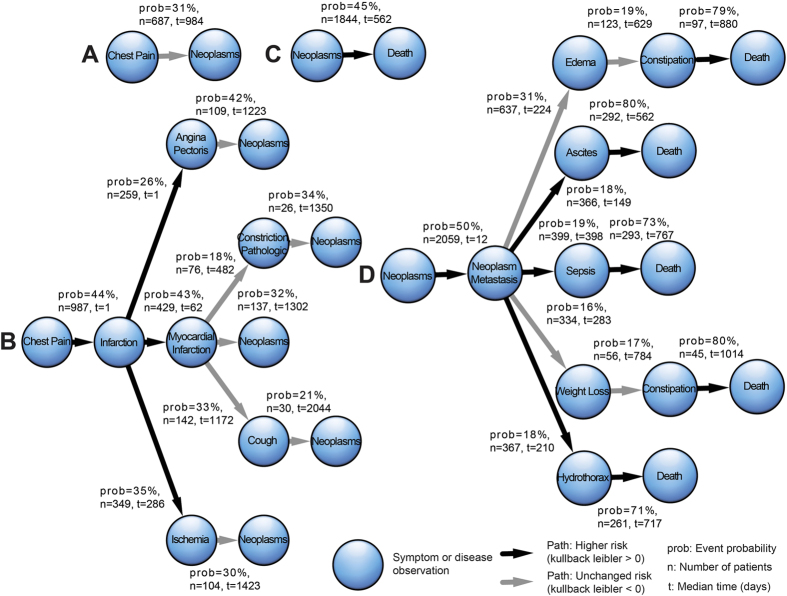
The most common symptoms and diseases reported prior to cancer diagnoses and from cancer to death. (**A**) The path from chest pain to cancer (neoplasms). (**B**) The paths from chest pain to cancer with intermediate events. (**C**) The path from cancer to death, and (**D**) the paths from cancer to death with intermediate events.

Among the 4,080 patients with cancer, 2,709 (66%) had events before the cancer diagnosis. Indeed, our patients had an average of 38 symptoms and diseases before the diagnosis of cancer with a median of 2.7 years. Supplementary Table S1 shows the list of symptom and disease events for our patients before cancer diagnoses. Among the total of 106 diseases and symptoms reported before the cancer diagnoses, 99 diseases and symptoms resulted in more than 10% of patients returning with cancer, 41 resulted in more than 20%, and three resulted in more than 30% (see an example for chest pain in Fig. 4A).

The five most informative trajectories with the highest information gain (KL-divergence)^28^ are presented in Fig. 4B. Among intermediate events, chest pain was associated with high risks of subsequent infarction (44%), myocardial infarction (43%), angina pectoris (42%) and ischemia (35%).

Figure 4C illustrates that 1,844 of the patients (45%) with cancer will die at a median of 1.5 years (562 days) after initial diagnosis. Considering the most informative trajectories from cancer to death, Fig. 4D illustrates that for 50% of the cancer patients, metastasis will be discovered within two weeks after the initial cancer diagnosis.

After the diagnoses of metastatic cancer disease, 18% of the patients will develop ascites with 80% experiencing subsequent death, 19% experiencing sepsis with 73% experiencing subsequent death and 18% experiencing hydrothorax with 71% experiencing subsequent death, 31% will develop edema, 16% weight loss, and patients with the subsequent observation of constipation have an 80% risk of death.

### Drawing causal conclusions and quantifying risks

**Figure 5:**
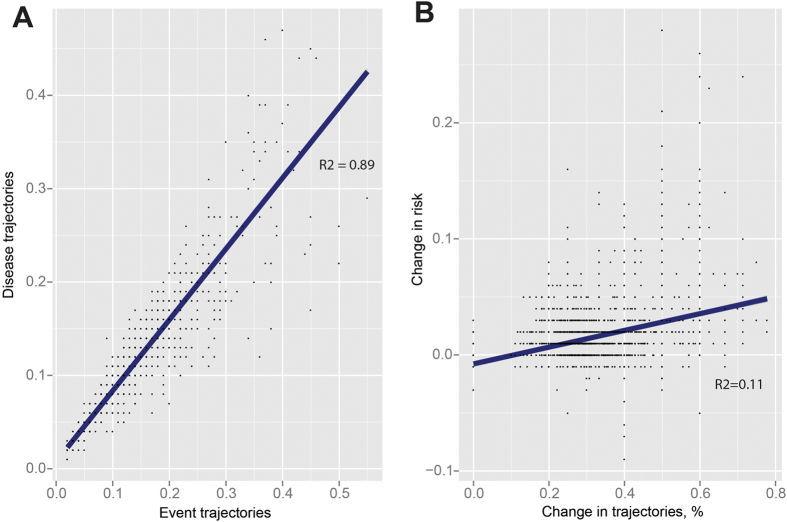
Control of confounding factors in event trajectories and disease trajectories. (**A**) The adjusted risks from the event trajectories on the x-axis and those of the disease trajectories on the y-axis correlate with the outcomes in the trajectories when controlling confounders. (**B**) The change in intermediate events and extraneous variables on the x-axis and the change in adjusted risk on the y-axis. The outcomes in the two sets remain the same when the intermediate events and extraneous variables change.

The risks adjusted by control of confounders for moving between events, symptoms and diseases are clearly desirable from a clinical perspective because they may disclose novel pathology. The two sets of trajectories, - disease trajectories and event trajectories - describe similar outcomes but with different extraneous variables. Figure 5A shows that the adjusted risks in the disease and event trajectories correlate (R2 = 0.89), while Fig. 5B shows that the variance between the disease trajectories and the event trajectories remains low (R2 = 0.11) with varying extraneous variables in the two trajectory sets.

**Figure 6:**
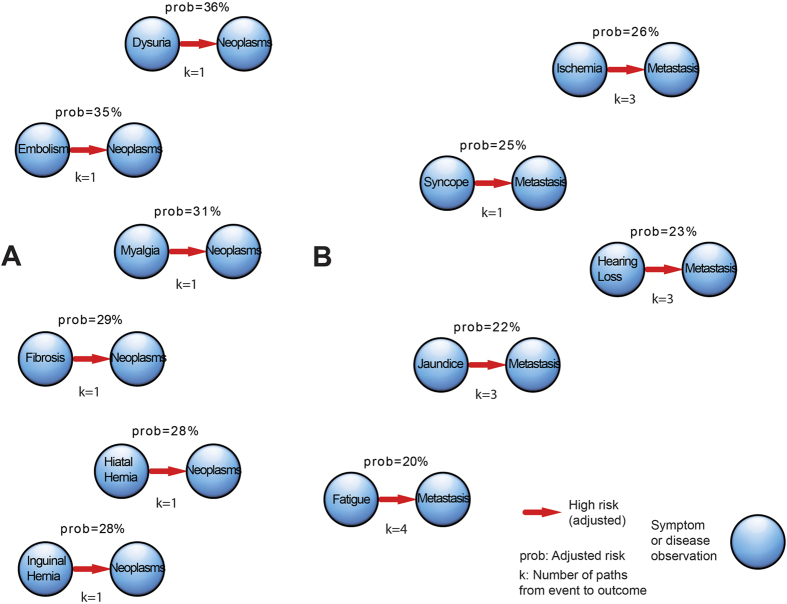
Adjusted risk of complication and readmission events. There are several paths that may bring a patient from an event to an outcome. Thus, we nominate the number of paths k. (**A**) The six events with high-risk for downstream complications and readmission with cancer. (**B**) The seven events with high-risk for downstream complications and readmission with metastasis.

Supplementary Table S2 presents a full list of adjusted risk markers for complications. From 8,326 quantified markers, we found 557 markers that constitute event risks higher than 20%. From high-risk markers, the most common were symptoms, such as vomiting (67 markers), nausea (41 markers), death (39 markers), hemorrhage (36 markers), metastasis (7 markers), and cancer disease (6 markers). The most common marker for the initial cancer diagnosis and subsequent metastasis are shown in Fig. 6A and B.

We identified dysuria (36% risk), embolism (35% risk), myalgia (31% risk), as high-risk markers for subsequent cancer disease. We identified ischemic disease (26% risk), syncope (25% risk), jaundice (22% risk), and fatigue (20% risk) as markers for metastatic cancer disease. The findings have been summarized in Table 1.

## Discussion

Despite decades of research, there is still a high risk of adverse events and mortality in modern cancer treatment, and many variables influence disease outcomes. To optimize treatment, and to prevent adverse events and hospital readmissions, patient related risk factors should be assessed continuously^29^,^30^. We believe our method of identifying patient trajectories through free text analyses may be used as a data-driven decision support tool during the complete cancer patient trajectory, to decrease adverse events and readmission, and to provide high quality cancer care.

With many risks factors that have to be recognized by the healthcare providers to reduce complications and provide high quality treatment^31^, the identification of the information in EHRs to support clinical decision-making is a labor-intensive task. To overcome this limitation, our method captures the massive unstructured text in the EHRs in a fully automated manner and infers patient trends from the available data. We may use these trends to place patients with longer medical histories and repeated readmissions on trajectories, from which data-driven clinical decisions can be made directly for the patient in real-time. For causality, we quantified risks via the control of confounders to provide a global risk assessment.

We demonstrate how to derive useful information from EHRs and how to control confounding factors in such data. We provide a full list of adjusted risks for progressing from symptoms to diseases and disclose a full list of risk factors for surgical patient outcomes. Moreover, we demonstrated for the first-time risk markers exhaustively quantified directly from text corpus of EHRs.

It is important to note that cancer patients are frail, with several concomitant chronic diseases, including chronic obstructive lung disease and coronary heart disease. As discussed by Reisinger et al., frailty among older cancer patients is increasingly recognized as a risk factor for decreased survival and poorer outcomes. Frailty is defined as a state of increased vulnerability toward stressors in older individuals, leading to an increased risk of developing adverse health outcomes^32^,^33^.

We believe our findings, as shown in Supplementary Tables S1 and S2, identify important stressors in older frail individuals leading to patient trajectories with a poorer outcome. The risk markers may assist in understanding outcomes and serve as early warnings to ensure high quality of care, and they may serve as starting points for further clinical elucidation. We believe that by understanding these risks and common patient trajectories, we may improve outcomes and increase patient safety.

The next step in this research is translational, i.e. how can we move this kind of statistical prediction tools from statistical laboratories to hospital departments. Clinical decision-making is challenging and is associated with tailored risk assessment. For cancer patients, common treatment questions are; Is it possible to tailor cancer treatment to the individual patient? Should this patient receive radio-chemotherapy? Should a tumor be surgically removed? What are the risks of adverse events after surgery? What is the risk of worsening of a chronic illness? Will surgical treatment improve survival? In general, these decisions are complex and are made in multidisciplinary team meetings. We believe that the proposed method may be used for real-time decision support in a multidisciplinary setting, to improve tailored cancer treatment. Importantly, the proposed methods, identifying individual cancer patient trajectories, may be used for shared decision-making where treatment options are discussed with the patient.

There exist limitations. Firstly: Observational data are by nature subject to selection bias because of information being recorded only for patients that already have a health problem, as in our case elderly patients undergoing surgery. Also, there is bias related to the most accurate information included in EHR, which means that we may be able to identify risk markers for patient outcomes, which may not directly infer causality. For example, chest pain was the most common symptom that was reported before the diagnosis of cancer, 31% (n = 687) of these patients were later diagnosed with cancer. We believe that this observation is explainable by confounding symptoms and diseases, as chest pain is not associated with cancer disease. When adjusting for confounders, the risk of moving from chest pain to cancer becomes negligible 4%. This illustrates the importance of understanding the difference between capturing data in EHRs and drawing causal conclusions because when capturing data directly in such observation data, the data is confounded.

Secondly, patient events in the EHRs constitute a scattered time space with no zero-time because of the health states of patients being in constant motion with multiple morbidities simultaneously. Shift registration is a known problem in data science, and methods do allow for time series curves to be fitted on irregularly sampled registration data^34^. However, these methods do not appear to apply to irregular sampled categorical data with repeated events and multiple morbidities. For this reason, observations and events reported for patients cannot be sequentially aligned. Jensen *et al*. 2014 described this problem, where the author’s condensed similar trajectories from structured diagnosis codes for the entire Danish population^10^. However, there are methods that have been found to be useful in exploring adverse drug effects, suicide risk, disease severity and patient stratification in EHR^35^,^36^,^37^,^38^,^39^,^40^, these methods depends strongly on the availability of structured information^41^,^42^,^43^,^44^.

Due to this, such methods are not easily translated to the clinical reality, and real-world applications are limited to work-flow discussions and documents searches^45^,^46^. A considerable obstacle to conceptualizing free text from EHRs is that the terms are not necessarily spelled accurately, and the sentences may not be grammatically correct. In some cases, the clinicians enter text directly into the records. This usually occurs during a consultation or immediately afterward. In other cases, the consulting physician records information on an audio-recorder and then the recording is transcribed later. In either case, the time and resources spent on entering text into the EHRs are sparse, and consequently, imbue the text corpus with a considerable amount of unstructured variation that cannot be handled linguistically.

With the clear majority of methods for processing text in EHRs being proprietary and those that are publicly available designed for English texts, we envision to provide with our method an open-source framework for the community that may be applied to and integrated into other existing EHR systems. We believe that the methodology presented here has the potential to improve health care and to provide consensus and transparency by taking the first steps towards standardized metrics that may be shared without privacy concerns between clinics. Also, clinicians and other healthcare providers may use the gained knowledge and develop methodologies to improve diagnoses applied in real-time on EHR data to identify cancer patients with high risk adverse events, decreased survival and a suboptimal patient trajectory.

## Methods

### Conversion of EHR text into conceptual information

The unstructured EHR text was conceptualized (annotated with the position of conceptual terms) by matching the terms and synonyms from the Norwegian version of the Medical Subject Headings^14^ (NOR-MeSH) to the text corpus of the EHRs.

We created a decompounder that splits compounded words into longest possible conceptual match to NOR-MeSH. We then manually evaluated the minimum length of splits that provided the most accurate decomposition of compound words from 1,000 random samples.

We allowed for some unstructured variations in the matching with the Smith-Waterman algorithm^19^ using a default setup in which match = 2, mismatch = −1 and gap = −1. Thus, one match weighs the same as two errors. The maximum number of gaps allowed in a match was subsequently manually evaluated in 1,000 random samples.

With a cutoff for the good alignments from the Smith-Waterman algorithm as a log-linear function of the length of the query string (corpus word) and the database string (NOR-MeSH term), the remaining fall-out (false positives) was addressed by manual curation of the top 2,500 most frequent terms in each MesH group (i.e., diseases, drugs and surgical procedures).

### Acquiring patient data in context

We trained Naïve Bayes classifiers to distinguish (1) real-time data, (2) retrospective data, and (3) negated findings and correspondences, such as internal communication (noise).

We created language features for each group as following: (i) the first 3 words before a paragraph with a matched term; this provides a feature for location of the term, (ii) the words in the paragraph of the term in which the term is used, (iii) the words in the sentence of the term; this provides features for the context in which the term is used, (iv) the 3 words before and after the term.

We then scored the language features with the tf-idf^20^,^47^ method and constructed a binary language feature vector for each MeSH group diseases, drugs and surgical procedures. The naïve Bayes classifiers were trained by labeling 1,000 randomly selected terms in each group (1), (2), (3) - one classifier for each group.

The performance of the classification was evaluated with 10-fold cross validation^48^. The features were included by sequential forward selection during each cross-validation cycle until the predictive performance stabilized; meaning no performance loss or gain was obtained with adding additional features.

Using the non-parametric Kolmogorov-Smirnov test, we evaluated whether the occurrences of symptoms and diseases among our patients depended on age^49^,^50^. This process was performed to evaluate whether age was a confounder when drawing trajectories. To correct for multiple testing, we use the Bonferroni method with a 25% level of significance to allow even smaller variations in age to be significant^25^.

### Construction of trajectories

We conducted Frequent Item Set (FIS) mining^27^ (Eclat/LCM) with a 5% minimum support and a minimum of two concepts for a set. We used the order of first appearance of events to perform a hierarchical count for the patients while proceeding from one event to the next. We condensed the trajectories with patient counts greater than 20 and calculated the probability of progressing one event on a trajectory as the fraction of the patients in an event node to the fraction of patients in the next event node. This procedure produces accurate probabilities for progressing on trajectories population wide, but the trajectories will not sum to one, as a patient can follow multiple trajectories that are not mutually exclusive. Time was measured as the median time for patients^51^ progressing from the first observation and event in the trajectory to the last. The construction of the trajectories was described previously in Fig. 2A–C: for a patient experiencing events *a, b, c* and *d*, we use trajectories with events *a* and *b* to predict *c,* and trajectories with *a, b* and *c* to predict *d*.

### Evaluation of trajectories and reconstruction of patient events

We evaluated the trajectories’ ability to correctly reproduce the patient data forward in time, using data from the 1,000 patients in our external data set, reserved prior to constructing the trajectories.

We evaluated the trajectories ability to reproduce the patient data by applying them step-wise forward on the patient data. The performance of the outcome predictions was compared to those of trajectories with a randomized event order of the same amount and sizes.

### Information scoring in trajectories

We scored the information carried by a trajectory using the Kullback-Leiber (KL) divergence^28^ as follows; to, from and between events we let *P(s*|*b*) be the measured probability of an event s and let *b* be the events observed prior to the event. If a patient experiences events, *a, b, c, d* and a second patient only *a, b, d*, the probability of *d* after the event *a* and *b* becomes 1. Thus, *a* and *b* are background events with *d* is outcome. The background probability *P(s*) was defined as the probability of randomly reaching an event *s* without considering the background events. In the above, simplified example the background probability of experiencing *c* is 0.5, while the background probability for *a, b* and *d* is 1.

Therefore, we defined the information gain in equation (1) for an event *s* in a trajectory as the KL-divergence between *P(s*|*b*) and the background *P(s*).


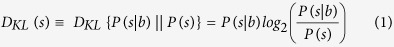


The information gain for the trajectory, *S*={*s*_*i*_|*i* = 1…*N*}, was defined as the sum of the contributions from each event, equation (2).


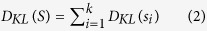


### Control of confounders in trajectories

The trajectories represent sequences of observations and events that are not all determinant of the outcome, and there may be several trajectories that can bring a patient from an event to an outcome. This is known as collider bias^52^. The non-causal associations may be classified into three categories; namely, inaccurate measurements (recall bias), selection bias and confounders^17^, where confounders are extraneous variables that correlate in the trajectories. For this reason, the trajectories depend on the preceding events, which act as confounding factors. There is no statistical test for confounders; however, we can provide an unbiased estimate of an outcome by controlling the confounders^53^,^54^,^55^.

Considering the events between event *x* and event *y* as confounding factors *z*, we have *N* trajectories, *S*_*n*_ = {*x, z*_*n*_*, y*} 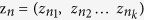, n = 1…N that will bring the patient from event *x* to event *y*.

An unbiased estimate *P(y*|*do(x*)) is obtained by averaging over the trajectories conditioned on the confounding factors, *do(x*), where the unbiased estimate *P(y*|*do(x*)) is formulated as equation (3).


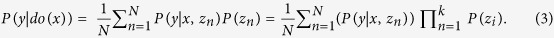


In equation (3) the outcome event *y* from event x is not considered confounded if *P(y*|*do(x*))=*P(y*|*x*)^53^.

## Additional Information

**How to cite this article:** Jensen, K. *et al*. Analysis of free text in electronic health records for identification of cancer patient trajectories. *Sci. Rep.* **7**, 46226; doi: 10.1038/srep46226 (2017).

**Publisher's note:** Springer Nature remains neutral with regard to jurisdictional claims in published maps and institutional affiliations.

## Supplementary Material

Supplementary Information

Supplementary Dataset S1

Supplementary Table S1

Supplementary Table S2

## Figures and Tables

**Table 1:** Number of temporal and high-risk events with a risk > 20%.

	Number of identified events	High-risk events
**Disease, symptoms and conditions**	8,326	557
	**Control of confounders**	**No control of confounders**
Risk correlation with and without confounder correction.	R2 = 0.89	R = 0.11
	**Diseases, symptoms, signs**	**Preceding events (n)**
Diseases, symptoms or signs with the highest number of preceding events	Vomiting	67
	Nausea	41
	Death	39
	Hemorrhage	36
	Metastasis	7
	Cancer	6
Diagnosis of cancer	**Preceding event**	**Risk, %**
	Dysuria	36
	Embolism	35
	Myalgia	31
Diagnosis of metastasis	**Preceding event**	**Risk, %**
	Ischemic disease	26
	Syncope	25
	Jaundice	22
	Fatigue	20

We evaluate the control of confounders by correlating the two trajectory sets with and without control of confounders and list the highlighted events together with cancer and metastatic risk events.
