# Supplementary material for: Analysis of free text in electronic health records for identification of cancer patient trajectories
Source: Sci Rep. 2017 Apr 7;7:46226. doi: 10.1038/srep46226 (PMC5384191; doi:10.1038/srep46226)
Supplement: Supplementary Information [file srep46226-s1.doc]

**Supplementary Material:**

**­­Analysis of free text in electronic health records for identification of cancer patient trajectories**

**Authors**

Kasper Jensen^1,2^, Cristina Soguero-Ruiz ^3^, Karl Oyvind Mikalsen ^4^, Rolv-Ole Lindsetmo ^5^, Irene Kouskoumvekaki ^6^, Mark Girolami ^2,7,8^, Stein Olav Skrovseth^1,4^, Knut Magne Augestad ^9^.

^1^Norwegian Centre for E-Health Research, University Hospital of North Norway, Norway

^2^Department of Statistics, University of Warwick, United Kingdom

^3^Department of Signal Theory and Communications, University Rey Juan Carlos, Spain

^4^Department of Mathematics and Statistics, UiT The Arctic University of Norway, Norway

^5^Department of Gastrointestinal Surgery, University Hospital of North Norway, Norway

^6^Department of Systems Biology, Technical University of Denmark, Denmark

^7^Department of Mathematics, Imperial College London, Exhibition Road, London SW7 2AZ

^8^The Alan Turing Institute, British Library, 96 Euston Road, London NW1 2DB

^9^Department of Gastrointestinal Surgery, Akershus University Hospital, Oslo, Norway

**Abstract**

With an aging patient population and increasing complexity in patient disease trajectories, physicians are often met with complex patient histories from which clinical decisions must be made. Due to the increasing rate of adverse events and hospitals facing financial penalties for readmission, there has never been a greater need to enforce evidence-led medical decision-making using available health care data. In the present work, we studied a cohort of 7,741 patients, of whom 4,080 were diagnosed with cancer, surgically treated at a University Hospital in the years 2004-2012. We have developed a methodology that allows disease trajectories of the cancer patients to be estimated from free text in electronic health records (EHRs). By using these disease trajectories, we predict 80% of patient events ahead in time. By control of confounders from 8326 quantified events, we identified 557 events that constitute high subsequent risks (risk>20%), including six events for cancer and seven events for metastasis. We believe that the presented methodology and findings could be used to improve clinical decision support and personalize trajectories, thereby decreasing adverse events and optimizing cancer treatment.

**Supplementary Figure S1**


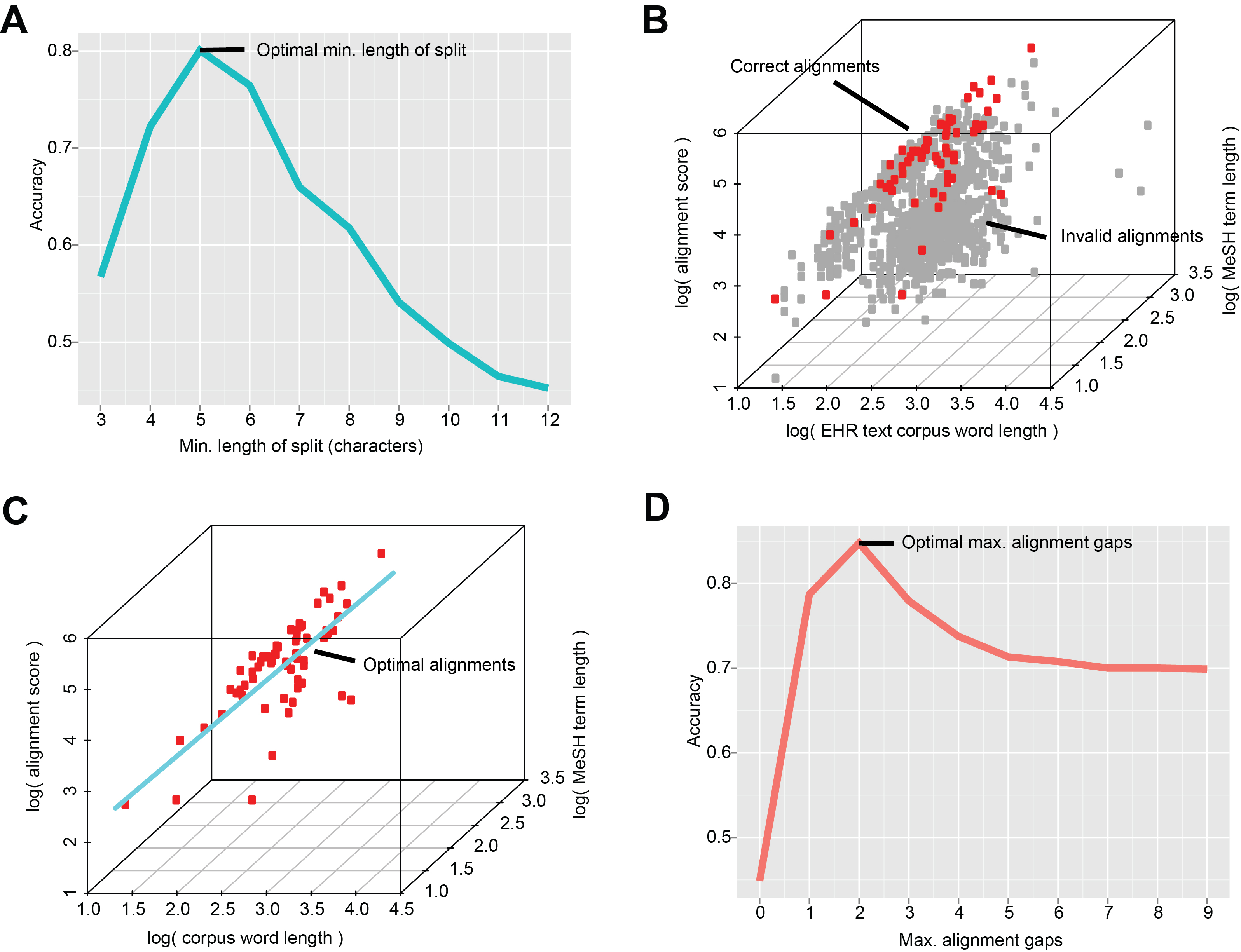


**Figure S1.** Evaluation of parameters for processing EHR text into conceptualized MeSH terms. **A)** Accuracy for the splits of compounded words and split length. **B)** Distribution of correct and invalid alignments in terms of log (EHR text corpus word length), log (MeSH term length) and log (alignment score). **C)** Cutoff scores for the alignments with the highest accuracies (optimal alignments). **D)** Alignment accuracy in terms of the maximum number of allowed gaps and maximum number of gaps with highest accuracy.

**Supplementary Figure S2**


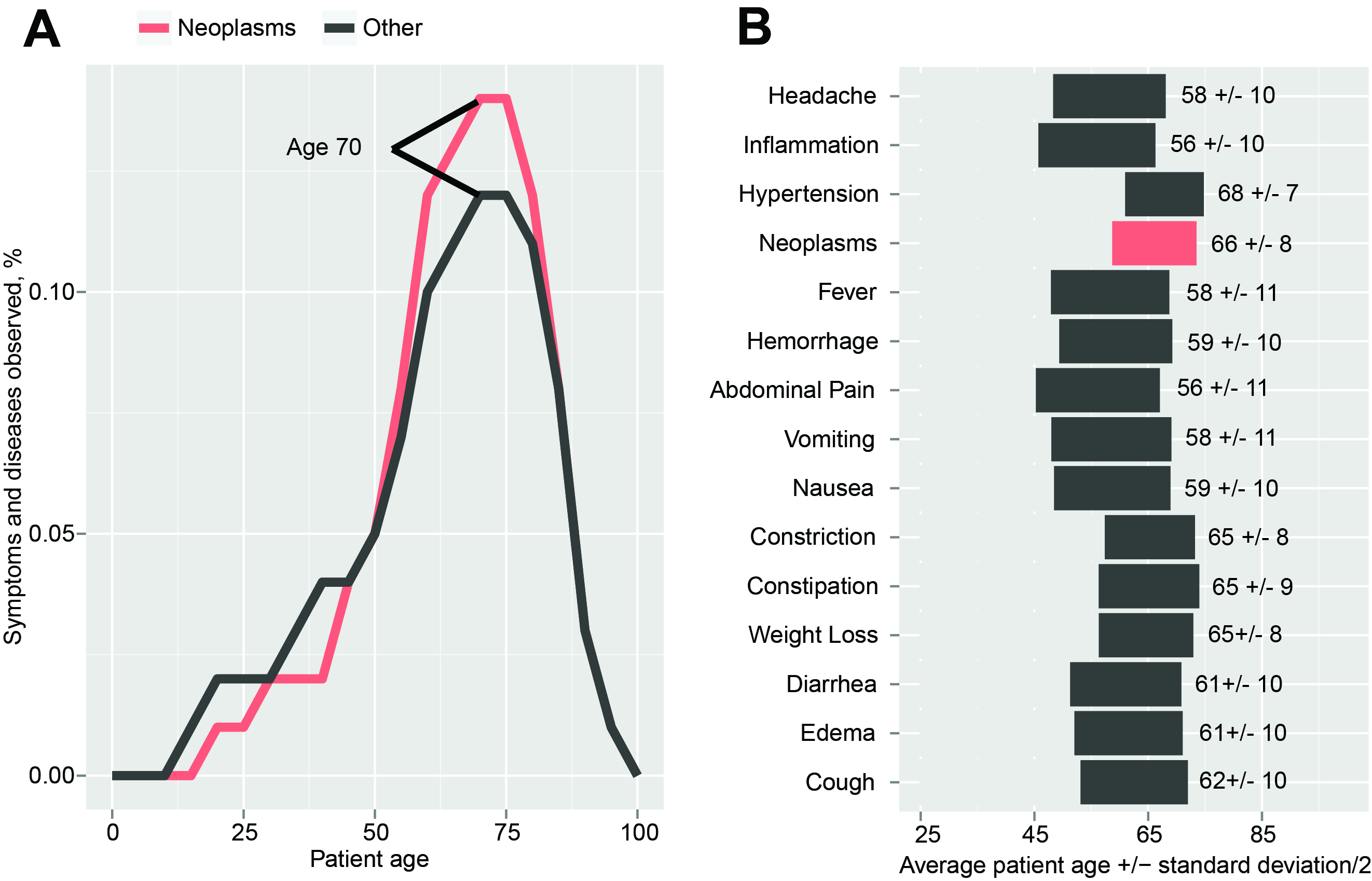


**Figure S2**. Symptom and disease distribution in relation to patient age. **A)** Distribution of cancer and other health problems. **B)** Symptoms and diseases with the highest variation in age, with the average patient age and standard deviation shown.
